# Long non-coding RNAs: potential new biomarkers for predicting tumor invasion and metastasis

**DOI:** 10.1186/s12943-016-0545-z

**Published:** 2016-09-29

**Authors:** Chunyang Jiang, Xin Li, Hui Zhao, Huibin Liu

**Affiliations:** 1 Department of Thoracic Surgery, Tianjin Union Medical Center, 190 Jieyuan Road, Hongqiao District, Tianjin, 300121 People’s Republic of China; 2 Tianjin Key Laboratory of Lung Cancer Metastasis and Tumor Microenvironment, Tianjin Lung Cancer Institute; Department of Lung Cancer Surgery, Tianjin Medical University General Hospital, 154 An Shan Road, Heping District, Tianjin, 300052 People’s Republic of China; 3 Department of pharmacology, Affiliated Tumor Hospital of Xinjiang Medical University, Urumqi, 830011 People’s Republic of China

**Keywords:** Long non-coding RNA, Cancer, Invasion, Metastasis, Biomarker

## Abstract

Long non-coding RNAs (lncRNAs) play important roles in malignant neoplasia. Indeed, many hallmarks of cancer define that the malignant phenotype of tumor cells are controlled by lncRNAs. Despite a growing number of studies highlighting their importance in cancer, there has been no systematic review of metastasis-associated lncRNAs in various cancer types. Accordingly, we focus on the key metastasis-related lncRNAs and outline their expression status in cancer tissues by reviewing the previous stuides, in order to summarize the nowadays research achivements for lncRNAs related to cancer metastasis. Medline, EMBASE, as well as PubMed databases were applied to study lncRNAs which were tightly associated with tumor invasion and metastasis. Up to now, a substantial number of lncRNAs have been found to have important biological functions. In this review, according to their various features in cancer, lncRNAs were roughly divided into three categories: promoting tumor invasion and metastasis, negative regulation of tumor metastasis and with dual regulatory roles. The present studies may establish the foundation for both further research on the mechanisms of cancer progression and future lncRNA-based clinical applications.

## Background

The Encyclopedia of DNA Elements (ENCODE) project, the functional annotation of all regulatory regions of the human genome, has confirmed that 80 % of the genome is transcribed into RNA but less than 2 % is translated into proteins. Those RNA molecules that lack protein-coding capability are collectively referred to as non-coding RNA (NcRNA) [1–3]. These NcRNAs are divided into housekeeping NcRNA and regulatory NcRNA [4], and, according to their molecular size, the latter can be subdivided into three major types: short NcRNA, mid-size NcRNA, and Long non-coding RNAs (lncRNAs) [5].

This review focuses on lncRNAs—those longer than 200 nucleotides (nt). Numerous studies have demonstrated that lncRNAs contribute to chromosome dosage-compensation, imprinting, epigenetic regulation, cell cycle control, nuclear and cytoplasmic trafficking, transcription, translation, splicing, and cell differentiation among other functions [6]. Studies also suggest that lncRNA aberrant expression is associated with numerous diseases including cancer. Currently, dozens of lncRNAs are implicated in the development and progression of cancer [7, 8]; hence, they may reveal novel mechanisms of transformation, tumor growth, and metastasis, as well as present new targets for cancer therapy.

## Biological functions of lncRNAs

In the following sections, we will highlight the gene regulatory mechanisms and signaling pathways dependent on lncRNAs. The majority of lncRNAs described were involved in regulating the expression of protein-coding genes in cis (affecting neighboring genes) or in trans (affecting distant genes on different chromosomes) [6, 8]. LncRNAs control transcription, translation, and protein function at multiple levels. Various lncRNAs have been implicated in the regulation of individual genes as well as gene expression programs through epigenetic regulation or by altering the basal transcriptional machinery. Specifically, lncRNAs can (1) interfere with downstream gene expression by transcription at the upstream promoter region of protein-coding genes; (2) affect the expression of multiple downstream genes by inhibiting the activity of RNA polymerase II or via chromatin remodeling and histone modification; (3) disturb mRNA splicing patterns so as to produce different splice variants by complementary binding with pre-mRNAs; (4) modulate protein activity through direct binding; (5) function as scaffolds to form RNA-protein complexes; (6) regulate the subcellular localization of specific proteins; (7) serve as transcriptional precursors of small RNAs that can also regulate gene expression [9]. Due to this functional diversity, it is reasonable to speculate that over- or underexpression of lncRNAs can compromise a myriad of biological processes, thereby contributing to the pathogenesis of disease.

In fact, there is now beyond doubt that lncRNAs are of clinical importance, including for cancer biology and treatment. Many lncRNAs exhibit altered expression levels in cancer cells compared with healthy tissue of the same origin [10, 11]. Utilizing comparative mammalian genomics approach coupled with evolutionary analysis, Khachane and Harrison identified a small population of conserved long non-protein-coding RNAs (lncRNAs) in the evolution, and these lncRNAs could play an important role in cancer pathomechanisms [12]. Here we will highlight recent studies supporting the direct involvement of individual lncRNA molecules in the invasion and metastasis of various types of cancer.

## LncRNAs in the invasion-metastasis cascade

Malignant tumors with infiltrative growth not only continuously grow and spread locally *in situ*, but also disseminate to other tissues through the lymphatic and circulatory systems as well as the body cavity via a process collectively referred to as tumor metastasis. Metastasis is one of the most significant biological characteristics of cancer and the leading cause of cancer-related death. Overexpression of NcRNAs are implicated in metastasis of human tumors, but most are shorter NcRNAs like microRNAs (miRNAs) [13]. However, recent studies have linked specific lncRNA gene mutations with cancer, raising the possibility of lncRNA-based cancer diagnostics and therapy. Dozens of lncRNAs are now widely believed to be involved in invasion and metastasis [14]. Therefore, this review will focus primarily on emerging mechanistic principles that underlie the nuclear functions of lncRNAs in order to provide oncologists with molecular insights that will help future research on lncRNAs in cancer management.

### LncRNAs promote tumor invasion and metastasis

For the few lncRNAs that are characterized at the functional level, evidence is accumulating that they play pivotal roles in malignant diseases. Many features that define the malignant cell phenotype are controlled by lncRNAs, particularly the processes involved in metastasis. Below we describe several lncRNAs implicated in the etiopathology of malignant disorders, particularly those that distinguish highly aggressive tumors from indolent forms (summarized in Table 1).

**Table 1:** LncRNAs with experimental data supporting the functions of promoting tumor invasion and metastasis

LncRNAs	Source (Tissues and/or cells)	Expression in patients	Related genes	Experimental data	Function	References
HOTAIR	Breast cancer, OSCC, GC, Nasopharyngeal carcinoma, ESCC, HCC, NSCLC, EOC, PCa, Colon cancer	Increased	PRC2, LSD1, H3K4, H3K27, HOXD10, PRG1, PCDH, WIF-1, Wnt/β-catenin signaling, MMP-9, VEGF, RBM38, EMT-related genes	Promote cell proliferation, increase invasion and metastasis, advanced clinical stage, bad prognosis	OG	[15–38]
MALAT1	Glioblastoma, Breast cancer, Lung cancer, ESCC, Pancreatic cancer, Kidney cancer, Bladder cancer, PCa, CRC, GC	Increased	WIF-1, N-Myc, JMJD1A, ATM-CHK2 pathway, p21 and p27, B-MYB, EMT-related genes, TGF-β, AKAP-9, SFPQ, PTBP2, Wnt/β-catenin signaling, miR-9, MEK/extracellular signaling	Increase tumorigenicity, promote cell proliferation, increase invasion and metastasis, advanced clinical stage, bad prognosis, inhibit cell cycle and apoptosis	OG	[39–57]
H19	GC, Ovarian cancer, Endometrial cancer, Bladder cancer, Epithelial cell	Increased	miR-675, ISM1, CALN1, let-7, HMGA2, c-Myc, Hmga2, Igf2bp3, EZH2, Wnt/β-catenin signaling, HGF/SF	Promote cell proliferation, increase invasion and metastasis	OG	[21, 58–64]
BANCR	Malignant melanoma, Lung cancer, Retinoblastoma, CRC	Increased		Promote cell proliferation, increase invasion and metastasis, advanced clinical stage	OG	[65–67]
CCAT1 & CCAT2	HCC, GC, Breast cancer	Increased	let-7, HMGA2, c-Myc, TPM1, BTG2	Promote cell proliferation, increase invasion and metastasis	OG	[68–70]
UCA1	TSCC, ESCC, CRC, Bladder cancer, Malignant melanoma	Increased		Promote cell proliferation, increase invasion and metastasis, inhibit cell cycle and apoptosis, advanced clinical stage, poor prognosis	OG	[56, 71–74]
FOXCUT	BLBC, OSCC, ESCC	Increased	FOXC1	Promote cell proliferation and colony formation, increase invasion and metastasis, advanced clinical stage	OG	[75–77]
AFAP1-AS1; HNF1A-AS1	EAC	Increased		Increase tumorigenicity	OG	[78, 79]
PEG10	Esophageal cancer cells	Increased		Inhibit apoptosis, increase invasion	OG	[80]
SPRY4-IT1; TUG1	ESCC	Increased	MAPK signaling pathway	Promote cell proliferation, increase invasion and metastasis, inhibit apoptosis, advanced clinical stage, poor prognosis	OG	[81–83]
SDMGC	GC	Increased	TRIM16	Promotes invasion and metastasis	OG	[84]
GAPLINC	GC	Increased	CD44	Promotes metastasis	OG	[85]
91H	CRC	Increased		Promotes migration, invasion and distant metastasis, poor prognosis	OG	[86]
ZXF1	Lung adenocarcinoma	Increased		Promotes migration, invasion and metastasis	OG	[87]
GHSROS; CARLo-5; AF118081	NSCLC	Increased		Promote cell proliferation, increase invasion and metastasis	OG	[88–90]
XIST	GSCs	Increased	miR-152	Promote cell proliferation, invasion, metastasis	OG	[91]
HIT; linc-ROR	Breast cancer	Increased	TGF-β signaling, EMT-related genes, certain miRNAs	Increase tumorigenicity, promote cell proliferation, increase invasion and metastasis	OG	[92, 93]
Loc554202	Breast cancer	Increased	LATS2, TNFAIP1	Promote cell proliferation, increase invasion and metastasis, inhibit apoptosis, advanced clinical stage	OG	[94]
BCAR4	Breast cancer	Increased	SNIP1, PNUTS, CCL21	Promotes cell metastasis	OG	[95]
RCCRT1	RCC	Increased		Promote invasion and metastasis, advanced clinical stage	OG	[96]
linc-UBC1; UCA1a (CUDR)	Bladder cancer	Increased		Promote tumorigenicity and colony formation, promote cell proliferation, increase invasion and metastasis, advanced clinical stage, bad prognosis	OG	[97, 98]
SChLAP1; linc00963; PCGEM1; PCAT18	PCa	Increased	EGFR signaling, p-AKT, miR-145, caspase 3/7	Promote tumorigenicity, promote cell proliferation, increase invasion and metastasis, inhibit apoptosis	OG	[99–102]
EBIC	Cervical cancer		EZH2, E-cadherin	Promote invasion and metastasis	OG	[103]
HOST2; TC0101441	Ovarian cancer	Increased	let-7b	Promote cell proliferation, increase invasion and metastasis	OG	[104, 106]
ZNF300P1	Ovarian cancer	Decreased		Promote cell proliferation and colony formation, increase metastasis	OG	[105]

*Abbreviations*: *OSCC* oral squamous cell carcinoma, *GC* gastric cancer, *ESCC* esophageal squamous cell carcinoma, *HCC* hepatocellular carcinoma, *NSCLC* non-small cell lung cancer, *EOC* epithelial ovarian cancer, *PCa* prostate cancer, *CRC* colorectal cancer, *TSCC* tongue squamous cell carcinoma, *BLBC* basal-like breast cancer, *OSCC* oral squamous cell carcinoma, *EAC* esophageal adenocarcinoma, *GSCs* glioblastoma stem cells, *RCC* renal cell carcinoma, *OG* oncogene

#### HOTAIR

*HOTAIR* is a non-coding 2.2-kb RNA gene located downstream, in the antisense direction, of the gene encoding homeobox C12 (*HOXC12*). The HOTAIR lncRNA acts as a scaffold for histone modification complexes, allowing them to coordinately interact with the histone modifiers Polycomb Repressive Complex 2 (PRC2, a histone methyltransferase) and lysine-specific demethylase 1 (LSD1). In turn, HOTAIR guides these proteins to specific genomic regions and regulates gene expression through histone tail methylation. In cancer cells, HOTAIR partners with PRC2 to induce genome-wide gene silencing. Enhanced expression of HOTAIR has been found in both primary and metastatic lesions of multiple cancer types. In most cases, elevated HOTAIR expression was correlated with metastasis and poor prognosis [15]. As a cancer-associated lncRNA, HOTAIR is a prospective biomarker of metastasis and poor prognosis in a diverse group of cancers.

HOTAIR facilitates H3 lysine 27 methylation by PRC2 in a fibroblast cell model (producing H3K27). In situ, this pathway facilitated the invasion-metastasis cascade of breast cancer by repressing the expression of hox transcription factor protein D10 (homeobox D10 or HOXD10), progesterone receptor 1 (PRG1), cell adhesion molecules protocadherins (PCDHs), and tumor angiogenesis-related molecular ephrin receptors [16]. Recent studies have confirmed that HOTAIR expression levels differ substantially between primary and metastasized breast cancer, and so could be used as a prognostic marker [17].

HOTAIR is also frequently upregulated in oral squamous cell carcinoma and nasopharyngeal carcinoma, and overexpression is highly correlated with tumor metastasis, clinical stage, and poor prognosis. Multiple lines of evidence have established that HOTAIR was involved in a large range of biological processes and diseases, particularly in cancer development and metastasis [18, 19].

HOTAIR expression is higher in gastric cancer tissues than corresponding noncancerous tissues. Expression level is significantly related to larger tumor size, advanced pathological TNM stage, and distant metastasis, as well as shorter patient survival rate, particularly when associated with lymph node metastasis. HOTAIR knockdown efficiently inhibits cell proliferation and matrix invasiveness in gastric cancer cells in vitro [20–23].

In 78 cases of esophageal squamous cell carcinoma (ESCC) patients, almost all with high expression of HOTAIR in tumor tissues (75/78, 96.15 %), expression level correlated with tumor metastasis, TNM staging, and lower overall survival (OS) rate. The five-year survival rate of patients with positive HOTAIR expression was greatly reduced compared with patients with negative expression. In vitro, HOTAIR facilitates ESCC cell proliferation, colony formation, and migration [24], while silencing of HOTAIR in ESCC KYSE30 cells reduced cell invasion and migration but enhances apoptosis rate [25]. In nude mice ESCC models, HOTAIR promoted cell proliferation and tumor metastasis, while knockout reduced the metastasis of ESCC cells [26]. HOTAIR may promote ESCC cell metastasis by inhibiting the expression of Wnt inhibitory factor 1 (WIF-1), thereby activating the Wnt/β-catenin signaling pathway [27].

Higher HOTAIR expression is also observed in hepatocellular carcinoma (HCC) tissues compared to adjacent non-tumor tissues, and again expression level is associated with lymphatic metastasis. Depletion of HOTAIR in the HCC cell line Bel7402 reduces expression levels of matrix metalloproteinase-9 (MMP-9) and vascular endothelial growth factor (VEGF), and consequently attenuates cell motility and metastasis [28]. Additionally, HOTAIR impedes RNA binding motif 38 (RBM38) protein expression and initiates HCC cell invasion and migration [29].

HOTAIR expression level is also elevated in metastatic lung cancer, and higher expression promotes tumor cell motility and invasion [30]. Of 77 cases of non-small cell lung cancer (NSCLC), 17 (22 %) exhibited high expression of HOTAIR, and these patients were more frequently in an advanced stage with lymph node metastasis or lymph-vascular invasion and had a shorter disease-free interval. Moreover, NSCLC cases with brain metastases showed higher HOTAIR levels [31]. Suppressing cellular HOTAIR by targeted RNA interference (RNAi) reduces NSCLC cell migration and invasion in vitro and prevents the metastases of cells in vivo [32].

In cervical cancer samples, overexpression of HOTAIR was associated with lymph node metastasis and shorter survival time [33]. Upregulation of HOTAIR can inhibit cell apoptosis, block the cell cycle, and accelerate cell growth, migration, and invasion, whereas HOTAIR downregulation has the opposite effects. Facilitation of matrix invasion and migration has been ascribed to HOTAIR-mediated activation of VEGF, MMP-9, and genes associated with epithelial-to-mesenchymal transition (EMT) [33] as well as inhibition of p21 [34]. The expression level of HOTAIR in endometrial cancer tissues is also higher than in normal endometrial tissues and is correlated with tumor stage, myometrium invasion, and lymph node metastasis. Reduction of HOTAIR level in endometrial cancer HEC-1A cells restrained cell proliferation, migration, and invasion [35]. Enhanced expression of HOTAIR in epithelial ovarian cancer (EOC) tissues was associated with clinical stage, pathological classification, lymph node metastasis, reduced OS, and shorter disease-free survival (DFS). Inhibition of HOTAIR in EOC cell lines (SKOV3. Ip1, HO8910-PM, and HEY-A8) in vitro depresses cell migration and invasion. The pro-metastatic effects of HOTAIR are partially mediated by the modulation of matrix metalloproteinases (MMPs) and EMT-related genes activities [36].

Compared to castration-sensitive prostate cancer (PCa) cells, HOTAIR expression is elevated in more aggressive castration-resistant PCa cells. A targeted short interfering transcript (siHOTAIR) reduced the viability and clonality of castration-resistant PCa and induced cell apoptosis and cycle arrest in vitro and in vivo [37].

Colon cancer is the most lethal archenteric cancer and is a common cause of cancer-related death worldwide. HOTAIR was overexpressed from 2- to nearly 1,600-fold in the cancer tissues. According to a HOTAIR expression level of >5-fold compared to adjacent tissues, 120 patients were divided into a high expression group (*n* = 40) and a low expression group (*n* = 80). Increased HOTAIR expression was strongly associated with depth of tumor invasion, lymph node metastasis, organ metastasis, pathological differentiation, vascular invasion, and tumor progression. Although the effect of HOTAIR on cell proliferation was modest, upregulation facilitated colon cancer initiation and progression [38].

#### MALAT1

Metastasis-associated lung adenocarcinoma transcript 1, (MALAT1, also known as NEAT2) is an lncRNA evolutionarily conserved across mammalian species, whereas no homolog is present in non-mammalian species. MALAT1 is involved in the formation of nuclear speckles, which are thought to be important for the processing of pre-mRNAs, and is further reported to be dysregulated in numerous cancers [39].

For glioblastoma, it cannot be completely resected due to its invasive characteristics, which makes it the most radical brain tumor in adults. MALAT1 is associated with EMT, which confers invasive capacity to malignant cells, and the depletion of MALAT1 can reduce the migration of glioblastoma cells. Furthermore, MALAT1 overexpression was reversed by WIF1-mediated attenuation of WNT signaling, which in turn inhibits glioblastoma cell migration [40]. Conversely, MALAT1 upregulation induced by N-Myc-activated JMJD1A gene expression enhances migration and invasion of glioblastoma cells [41].

In breast cancer cell lines MCF10a, MCF7, and MB231, levels of MALAT1 were significantly decreased by treatment with 100 nM 17β-Estradiol (E2), and the E2 treatment affects breast tumor cells proliferation, migration and invasion in an ERα -independent, but a dose-dependent way by decreasing the MALAT-1 RNA level [42].

In lung cancer, MALAT1 was initially discovered as a predictive biomarker for metastasis that induces the expression of metastasis-associated genes. Depletion of MALAT1 in the adenocarcinomic human alveolar basal epithelial cell line A549 decreased cell migration, increased apoptosis, and inhibited clonogenic growth. Similarly, diminished MALAT1 expression impaired tumor formation and growth in nude mice [43, 44].

Upregulation of MALAT1 was also highly correlated with clinical stage, primary tumor size, and lymph node metastasis of ESCC in vivo and in vitro [45]. In one series, high MALAT1 expression was observed in 46.3 % of ESCCs. Over-expression of MALAT1 was most frequently in advanced stage tumor samples. MALAT1 affected cell growth through regulation of the ATM-CHK2 pathway, allowing gene amplification in the tumor progression period. Post-transcriptional silencing of MALAT1 can reduce ESCC cell proliferation through cell cycle block, and this may relate to suppression of MALAT1-mediated upregulation of p21 and p27 as well as inhibition of the cell cycle-associated transcription factor B-MYB. In addition, knockdown of MALAT1 enhanced resistance to ESCC cell invasion and metastasis [46].

MALAT1 expression was also higher in pancreatic tumor specimens than in matched normal tissues and was upregulated in seven pancreatic cancer cell lines [47]. Stable knockout of MALAT1 prevents cell proliferation and motility, which is associated with G2/M arrest, enhanced apoptosis, disrupted EMT, and weakened cancer stem-like properties in vitro, consistent with a putative oncogenic role for MALAT1 in this usually fatal disease [47].

MALAT1 is upregulated in a number of urologic neoplasms. Higher MALAT1 expression was detected both in clear cell renal cell carcinoma (ccRCC) tissues than in adjacent non-tumor tissues and also in kidney cancer cells than normal epithelial HK-2 cells. The ccRCC patients with higher MALAT1 level more often had advanced clinical features (such as greater tumor size, depth of tumor invasion, and lymphatic invasion) and poor survival rate [48]. MALAT1 is also upregulated in bladder cancer tissues relative to matched non-cancerous tissue samples. Knockout of MALAT1 in bladder cancer animal models resulted in inhibition of malignant cell metastasis by reducing Wnt signaling and thereby suppressing EMT [49, 50].

Overexpression of MALAT1 is observed in human PCa tissues and cell lines and is closely associated with high patient Gleason score, prostate specific antigen expression, clinical stage, and castration resistant PCa. Silencing of MALAT1 abolished cell proliferation by decelerating the G0/G1 phases of the cell cycle in castration-resistant PCa cells. In castrated male nude mice, siRNA-mediated silencing of MALAT1 delayed tumor growth and inhibited PCa cell metastasis [51].

A functional study showed that the 3′ end of MALAT1 played an important role in metastasis of colon cancer and colorectal cancer (CRC) [52]. MALAT1 is highly induced in human CRC tissues *in situ* and often accompanies lymph node metastasis. Overexpression of MALAT1 in mice using a lncRNA gain-of-function system promoted CRC cell proliferation, invasion, and migration, and enhanced tumor growth and metastasis after implantation. MALAT1 also exhibited positive effects on CRC cell motility in vitro. The MALAT1 target gene PRKA kinase anchor protein 9 (AKAP-9) was highly expressed in both CRC cells and tissues with metastasis potential. MALAT1 may thus promote invasion and metastasis by targeting AKAP-9 [53]. Additionally, MALAT1 can release proto-oncogene PTBP2 by combining with the tumor suppressor gene SFPQ, resulting in CRC metastasis and disease progression [54]. Resveratrol, extracted from the Chinese herbal medicine *Polygonum cuspidatum*, can downregulate MALAT1 and attenuate Wnt/β-catenin signaling, resulting in enhanced resistance to invasion and metastasis of human CRC [55].

MALAT1 has also been implicated in post-transcriptional gene regulation. In osteosarcoma (OS) MG-63 cells treated with high-dose E2, MALAT1 bound and sequestered miR-9, thereby indirectly inducing genes normally suppressed by miR-9. Decreasing the level of MALAT1 attenuates EMT [56]. Furthermore, high expression of MALAT1 was found exclusively in metastatic tissues developed in lymph node, and knockout of MALAT1 inhibits melanoma cell migration in vitro [57].

MALAT2 is an lncRNA similar to MALAT1 in structure and function. In 146 cases of stage II/III gastric cancer (GC), increased expression of MALAT2 was positively correlated with tumor stage and lymph node metastasis. Ectopic expression of MALAT2 enhanced the motility of the human GC cell line SGC-7901 in vitro, whereas silencing of MALAT2 impairs tumor metastasis. MALAT2 may function by inducing EMT via the MEK/extracellular signal-regulated kinase signaling pathway [58].

#### H19

H19 is a paternally imprinted gene located at chromosome 11p15.5 that was widely studied in cancer biology even before lncRNAs had gained the attention of cancer researchers. H19 is re-expressed during tumorigenesis and promotes tumorigenic properties in multiple tissues, including stomach, ovary, endometrium, and bladder. Overexpression of H19 was believed to contribute to cell proliferation and viability of GC cells through the upregulation of miR-675, which resulted in the induction of the angiogenesis inhibitor ISM1 and calcium-binding protein CALN1 genes [21, 59–61]. The oncogenic properties of H19 were strongly associated with antagonism of the tumor suppressor miRNA let-7 and forced EMT mediated by the non-histone chromosomal transcriptional regulator HMGA2. Suppression of H19 using a targeted siRNA could inactivate EMT [62]. MicroRNA let-7 reduces cell growth and motility through post-transcriptional suppression of oncogenes. In ovarian and endometrial cancer, the reciprocal functions of H19 and let-7 were associated with let-7-mediated regulation of metastasis-related genes, including c-Myc, HMGA2, and IGF2BP3 [63].

H19 level is elevated in bladder cancer tissues, and upregulated expression of H19 promoted cell motility and metastasis of bladder cancer cells in vivo and in vitro. H19-regulated malignant properties were associated with enhancer of zeste homolog 2 (EZH2), the catalytic subunit of PRC2. In this case, malignancy associated with H19 appeared to be mediated by activation of Wnt/β-catenin and E-cadherin signaling [64].

Moreover, H19 is overexpressed in epithelial cells and is a target gene of fibroblast-derived growth factor HGF/SF, a known regulator of epithelial − mesenchymal interactions, implicating H19 upregulation in cell morphogenesis and migration [65].

#### Bancr

The lncRNA BRAF-activated non-coding RNA (BANCR) has been implicated in the initiation and progression of malignant melanoma and lung cancer. BANCR overexpression is observed in melanoma cells and is significantly associated with metastasis. Transfection of a targeted BANCR siRNA resulted in diminished invasive and migratory potential compared to cells transfected with control siRNA [66]. In retinoblastoma tissues, high BANCR expression was correlated with tumor size and invasion of choroid and optic nerve. Retinoblastoma cells transfected with siBANCR exhibit reduced colony formation compared to siControl-transfected cells in vitro [67].

In 60 cases of CRC, BANCR expression was upregulated compared to corresponding normal specimens. Furthermore, high expression was correlated with poor prognoses. Ectopic expression of BANCR was demonstrated in CRC Caco-2 and HCT116 cells, and BANCR silencing inhibits the migratory potential of these cell lines compared to mock-transfected cells [68].

#### CCAT1 & CCAT2

Colon Cancer Associated Transcript 1 (CCAT1) is a newly discovered lncRNA that has been characterized in detail by RNA disruption assay (RDA), cDNA cloning, and rapid amplification of cDNA ends (RACE). It likely participates in the genesis, development, invasion, and metastasis of CRC, and so has garnered intense research interest. Moreover, in a study of 66 HCC cases, expression levels of CCAT1 were dramatically higher in cancer tissue than matching normal liver samples. Further research supported the notion that CCAT1 could promote invasion and metastasis of HCC by competitive combination with let-7 and inhibition of HMGA2 and c-Myc, both endogenous target genes of let-7 [69]. Likewise, CCAT1 expression is higher in primary GC tissues than in adjacent normal mucosa. A chromatin immunoprecipitation assay showed that c-Myc could directly combine with the E-box element of the CCAT1 promoter region, thereby increasing CCAT1 expression. Consequently, CCAT1 overexpression enhances the growth and invasive capacities of GC cells [70].

CCAT2 was upregulated in two thirds of a large cohort of primary breast cancer patients (997) and was a significant risk factor for distant metastases [71]. CCAT2 was also reported to be associated with colon cancer. It is highly overexpressed in microsatellite-stable CRC and promotes tumor growth, metastasis, and chromosomal instability. Upregulated Myc expression and WNT signaling induced by CCAT2 contributes to CRC pathogenesis [72].

#### UCA1

Urothelial carcinoma-associated 1 (UCA1), an oncofetal gene involved in embryonic development and carcinogenesis, often exhibits extraordinarily high expression in tumor tissues and cancer cells. In 94 cases of tongue squamous cell carcinoma (TSCC), expression of UCA1 was much higher than in adjacent normal samples. As expected, the ectopic expression of UCA1 induced a marked increase in lymph node metastasis [73].

Elevated levels of UCA1 are also found in ESCC tissues and the immortalized esophageal epithelial cell line NE1 compared to respective controls. UCA1 overexpression was often accompanied by tumor metastasis, suggesting that this lncRNA could be clinically useful as a diagnostic and prognostic indicator for ESCC patients [74].

In CRC tissues, high levels of UCA1 expression are correlated with larger tumor volume, greater invasion depth, less differentiated histology, and shorter survival. These results implicate UCA1 in the pathogenesis of CRC by enhancing the rates of cell growth, colonogenic survival, and the invasive and migratory potential of CRC cells [75]. Furthermore, under hypoxia, UCA1 levels are enhanced in bladder cancer cells compared to controls, and this high level of UCA1 expression is significantly correlated with greater tumor depth and apoptosis escape [76].

Expression of UCA1 is also higher in advanced stage (III/IV) compared to early stage (I/II) melanoma. UCA1 upregulation was correlated with poor differentiation, advanced lymph node classification, and metastasis of melanoma cells, while invasive and migratory capacities were remarkably diminished by UCA1 knockdown [57].

#### FOXCUT

FOXC1, a member of the Forkhead Box (FOX) family of transcription factors, is a key regulator of tumor occurrence and progression. The lncRNA-mRNA pair FOXCUT − FOXC1 may be a new functional form. Recent studies have demonstrated that high expression levels of FOXCUT and FOXC1 are strongly associated with poor prognosis in patients with base-like breast cancer (BLBC). Conversely, inhibition of FOXCUT impaired the invasion and migration capabilities of the breast cancer cell lines MDA-MB-231 and MDA-MB-468 [77].

A recent report indicated that FOXCUT was also more highly expressed in oral squamous cell carcinoma (OSCC) tissues compared to matching adjacent normal specimens and suggested a positive correlation with FOXC1. Transfection of Tca8113 and SCC-9 OSCC cell lines with a FOXCUT siRNA resulted in a substantially reduced number of colonies and lower migratory potential [78]. Furthermore, FOXCUT overexpression level was associated with tumor stage, metastasis, and post-operative survival of patients with ESCC. FOXCUT silencing in vitro using siRNA inhibited ESCC cell growth, colony formation, and dissemination compared to mock-transfected cells [79].

### Other lncRNAs upregulated in tumor metastasis

#### Esophageal adenocarcinoma and esophageal squamous cell carcinoma

Expression levels of the lncRNAs AFAP1-AS1, HNF1A-AS1, and PEG10 are all markedly higher in malignant esophageal adenocarcinoma (ECA) tissues than in normal para-carcinoma samples, and expression correlates with cell motility, local infiltration depth, and vascular invasion as well as with tumor staging and differentiation level. On the contrary, knockdown of these lncRNAs lead to diminished cell viability and increased anoikis in EAC EC9706 and KYSE150 cells compared to cells transfected with empty vector [80–82].

Increased levels of the lncRNAs SPRY4-IT1 and TUG1 are observed in ESCC tissues and several ESCC cell lines. High SPRY4-IT1 expression level is positively correlated with FIGO stage, histological tumor grade, and lymph node metastasis. Suppression of the SPRY4-IT1 or TUG1 gene reduces invasive ability of ESCCs by blocking progression of the cell cycle [83, 84]. Khaitan et al. [85] found that local infiltration depth of neuroblastoma was correlated with dysregulated expression of SPRY4-IT1, leading to repressed proliferation and extensive apoptosis. This effect was mediated by multiple MAPK signaling molecules, including Raf1, B-Raf, MEK1/2, TESK1, MARKK, and MARK2. Thus, greater understanding of the underlying mechanisms of SPRY4-IT1 in the molecular etiology of ESCC could lead to major advances in lncRNA-directed diagnostics and define new therapeutic targets against this disease.

#### Gastric cancer and colorectal cancer

The lncRNA SDMGC (special for distant metastasis of GC) is an indicator of poor survival rate whilst its target gene TRIM16 is a positive prognostic factor in GC. Inhibition of SDMGC by RNAi decreased invasion of GC cells while suppression of TRIM16 has the opposite effect [86]. The lncRNA GAPLINC (gastric adenocarcinoma predictive long intergenic noncoding RNA) is also upregulated in GC patients, particularly with stage IV and distant metastasis. In addition, GAPLINC can function as a tumor promoter by upregulating CD44, a cell surface glycoprotein known to facilitate tumor metastasis [87].

Lately, it has been reported that lncRNAs regulated by transforming growth factor (TGF)-β (ATB) could initiate the invasion-metastasis cascade in CRC. Forced expression of ATB was closely related to bigger tumor volume, depth of tumor invasion, vascular and lymphatic invasion, as well as lymph node metastasis. In one study, patients with high ATB expression had strikingly shorter overall survivals than those with low expression [88]. Furthermore, higher ATB expression was detected in cancer patients with the transfer of blood-brone. Thus, ATB may be involved in CRC progression and could develop a promising indicator of worse prognosis [88].

#### Lung cancer

Lung cancer is the leading cause of cancer-related death and remains a major public health problem worldwide. Aside from HOTAIR and MALAT1, several other lncRNAs are correlated with lung cancer metastasis. LncRNA ZXF1 expression is markedly higher in lung adenocarcinoma tissues compared to adjacent non-cancerous lung tissues, and upregulated ZXF1 is correlated with the presence and extent of lymph node metastasis as well as tumor pathological stage. The three-year OS rate of patients with higher ZXF1 expression is reduced compared to patients with lower ZXF1, implying that high ZXF1 expression could be a marker for poor prognosis. In vitro, knockdown of ZXF1 by siRNA depressed the invasion and migration of A549 cells, whereas no remarkable effect was observed on cell growth [89].

LncRNA GHSROS (GHSR opposite strand) is transcribed from the antisense strand of the ghrelin receptor gene growth hormone secretagogue receptor (GHSR). Engineered overexpression of GHSROS stimulated cell migration in the A549 and NCI-H1299 NSCLC cell lines, but suppressed cell migration in the normal lung-derived bronchoepithelial cell line Beas-2B, indicating that GHSROS function may be dependent on the oncogenic context [90].

Cancer-associated region long non-coding RNA (CARLo-5) initially characterized in colon cancer was identified a fresh lncRNA. Subsequent studies have documented marked upregulation of CARLo-5 in NSCLC tissues compared to adjacent normal specimens. Moreover, patients with higher CARLo-5 expression levels have considerably worse prognosis than those with low expression. In vitro experiment, knockdown of CARLo-5 inhibited the proliferative activity, infiltration, and migration of NSCLC cell lines. Additionally, inhibition of CARLo-5 could reverse EMT in a NSCLC cell line. In summary, these results indicated that CARLo-5 may serve as a new prognostic marker and a potential molecular target in treatment of NSCLC [91].

LncRNA AF118081, a new oncogenic lncRNA, is the most highly overexpressed lncRNA in the transformed bronchial epithelial cell line 16HBE-T as well as in lung cancer specimens, while knockdown in 16HBE-T cells inhibited proliferation and invasion. In agreement with the results of in vitro assays, downregulation of AF118081 in a xenograft mouse model suppresses tumor growth [92].

#### Glioblastoma multiforme (GBM)

Glioblastoma multiforme (GBM) is the most common and aggressive primary brain tumor. The lncRNA XIST (X-inactive specific transcript) is the master regulator of X inactivation in mammals. XIST expression is upregulated in glioma tissues and human glioblastoma stem cells (GSCs), while knockdown of XIST reduced cell proliferation, migration, and invasion, and induced apoptosis. In vivo knockdown of XIST restrained tumor growth and produced higher survival in nude mice. XIST could mediate its oncogenic effect at least in part by binding the tumor suppressor miR-152 [93].

#### Breast cancer

Commandeering of EMT regulatory pathways is a common mechanism whereby tumor cells depart from the primary site to invade surrounding tissue and establish distant metastases. Transforming growth factor beta (TGF-β) signaling is a major decoy of EMT capable of facilitating breast cancer metastasis, a process modulated by the lncRNA HIT (HOXA antisense transcript induced by TGF-β). HIT expression is dramatically increased in the highly metastatic 4 T1 cell line, and knockout of HIT in 4 T1 cells resulted in decreased cell migration and invasion. Moreover, higher HIT expression was associated with more invasive human breast carcinoma in patients [94].

Linc-ROR is a large intergenic non-coding RNA of about 2600 nt first described in induced pluripotent stem cells (iPSCs) and as a regulator of embryonic stem cell generation. Ectopic overexpression of linc-ROR enhanced breast cancer cell migration and invasion, whereas silencing repressed tumor growth and lung metastasis in vivo. Mechanistically, linc-ROR was associated with miRNPs (ribonucleoprotein complexes containing multiple miRNAs) and acted as a competitive endogenous RNA to miR-205, a member of the miRNA-200 family frequently suppressed in high-grade tumors. Specifically, linc-ROR prevented the degradation of miR-205 target genes, including the EMT inducer ZEB2. Thus, linc-ROR can operate as a chief regulator of EMT and advance the progression and metastasis of breast cancer through regulation of miRNAs [95].

The lncRNA Loc554202 gene at 9p21.3 does not contain an open reading frame, but rather is transcribed from four exons to create a spliced transcript of 2.2 kb. Loc554202 is expressed at markedly higher levels in breast cancer tissues than in normal controls and is associated with advanced pathologic stage and greater tumor size. Knockdown of Loc554202 depressed breast cancer cell proliferation, induced apoptosis, and inhibited migration/invasion in vitro as well as tumorigenesis in vivo [96].

LncRNA BCAR4 regulates a number of developmental and tumorigenic processes by activating a non-canonical Hedge-hog/GLI2 transcriptional program that promotes cell migration. Increased BCAR4 level was correlated with progressive mammary cancer, and targeting BCAR4 based on knockout of specific gene intensively suppressed breast cancer spread in an animal model [97].

#### Urologic neoplasms

The lncRNA RCCRT1 is upregulated in renal cell carcinoma (RCC) compared to adjacent noncancerous tissues, particularly in high-grade RCC tissues. Furthermore, siRNA-induced depletion of RCCRT1 expression diminished migration and invasion in ACHN and A498 RCC cell lines [98].

The lncRNA linc-UBC1a (up-regulated in bladder cancer 1a) was aberrantly expressed in a large cohort (103 cases) of bladder cancer and correlated with poor prognosis. Knockdown of linc-UBC1a decelerated the growth rate of bladder cancer cells, resulting in decreased tumor growth and metastasis [99]. LncRNA linc-UBC1a is identical to the previously reported cancer-upregulated drug resistant (CUDR) gene, which is known to play a pivotal role in both embryonic development and bladder cancer progression. Overexpression of UCA1a/CUDR elevated proliferation, migration, and invasion of the bladder cancer cell line UM-UC-2 in vitro [100].

SChLAP1 is abundantly expressed in approximately 25 % of prostate cancers and aids in the discrimination of aggressive tumors from indolent forms of the disease. SChLAP1 can coordinate cancer cell invasion in vitro and metastatic spread in vivo by impairing SNF5-mediated regulation of gene expression and genomic binding [101].

Linc00963 is upregulated in androgen-dependent LNCaP and androgen-independent C4-2 cell lines. Knockdown of linc00963 attenuated the expression of EGFR and the phosphorylation level of AKT even promoted apoptosis in C4-2 cells. These results suggested that Linc00963 prompted PCa transition from androgen-dependent to androgen-independent and metastasis via the EGFR signaling pathway [102].

The lncRNA PCGEM1 (Prostate cancer gene expression marker 1) has drawn increasing attention for its important role in PCa. PCGEM1 polymorphisms may contribute to PCa risk in Chinese men [103]. LncRNA PCAT18 is specifically expressed in PCa, and PCAT18 silencing significantly inhibited PCa cell proliferation, migration, and invasion, and triggered caspase 3/7 activation with no effect on non-neoplastic cells [104].

#### Female reproductive system tumors

LncRNA EBIC is another oncogenic lncRNA that regulates metastasis of cervical cancer by binding to the transcription repressor EZH2, thereby inhibiting E-cadherin expression [105]. LncRNA HOST2 (human ovarian cancer-specific transcript 2) is specifically overexpressed in human ovarian cancer. HOST2 inhibited miRNA let-7b function by reducing it bioavailability, leading to post-transcriptional suppression of oncogenes that regulated cell growth and motility [106]. The novel lincRNA ZNF300P1 is frequently hypermethylated in multiple ovarian cancer tissues and cell lines, and its expression can influence cell polarity, motility, and adhesion, while loss of expression may contribute to the metastatic potential of ovarian cancer cells [107].

EOC tissues expressing E2 receptor alpha (ERα) also express higher levels of lncRNA TC0101441 compared to ERα-negative tissues. Ectopic TC0101441 expression was correlated with lymph node metastasis, while knockdown impaired E2-induced EOC cell migration/invasion [108].

In aggregate, these findings implicate multiple lncRNAs as possible diagnostic and therapeutic targets for aggressive and metastatic cancers. In particular, the antitumor effects of targeted knockdown highlight the potential of lncRNA-based cancer therapies for patients at high risk for metastasis, an outcome currently lacking effective chemotherapeutic options.

### LncRNAs involved in negative regulation of tumor metastasis

Compared to oncogenic lncRNAs, few lncRNAs have been confirmed as negative regulators of tumor invasion and metastasis. Such anti-tumor lncRNAs are discussed below along with their correlated expression in tumors (summarized in Table 2).

**Table 2:** LncRNAs with experimental data supporting the functions of inhibiting tumor invasion and metastasis

LncRNAs	Source (Tissues and/or cells)	Expression in patients	Related genes	Experimental data	Function	References
BANCR	SCLC cell lines	Decreased	p38 MAPK, JNK	Inhibit cell proliferation, decrease invasion and metastasis	TS	[107]
DRAIC; PCAT29	PCa			Inhibit tumorigenicity and cell proliferation, decrease invasion and metastasis	TS	[14, 108]
TSLC1-AS1; ADAMTS9-AS2; CASC2	Glioblastoma, Glioma	Decreased	DNMT1, miR-21	Inhibit cell proliferation, decrease invasion and metastasis, induce cell apoptosis	TS	[109–111]
LCE5A-1; KCTD6-3	HNSCC	Decreased	EMT related genes	Reduce cell proliferation and metastasis, associated with the prognosis	TS	[112]
GAS5; CADM1-AS1	RCC, ccRCC	Decreased	CADM1	Inhibit cell proliferation and cell growth, decrease migration and invasion, induce apoptosis and cell cycle arrest	TS	[113, 114]
PTENP1	HCC	Decreased	PI3K/AKT pathway	Inhibit tumor growth and cell proliferation, cell invasion and migration, induce cell autophagy and apoptosis	TS	[115]
FENDRR	GC	Decreased	FN1, MMP2, MMP9	Inhibit cells invasion and migration	TS	[116]
ENST00000480739; Meg3	PDAC, PNETs	Decreased	c-Met	Prevent cell proliferation and delay cell cycle, decrease cell migration, invasion and metastasis	TS	[117, 118]

*Abbreviations*: *SCLC* small cell lung cancer, *PCa* prostate cancer, *HNSCC* head and neck squamous cell carcinoma, *RCC* renal cell carcinoma, *ccRCC* clear cell renal cell carcinoma, *HCC* hepatocellular carcinoma, *GC* gastric cancer, *PDAC* pancreatic ductal adenocarcinoma, *PNETs* pancreatic neuroendocrine tumors, *TS* tumor suppressor

#### Lung cancer

In contrast to findings implicating BANCR in malignant melanoma, lung cancer, and CRC metastasis, BANCR levels are downregulated in NCI-H1688 and NCI-H446 lung carcinoma (LC) cell lines. When BANCR expression was strengthened by gain-of-function, tumor growth depressed and vice versa. In addition, BANCR was found to regulate LC proliferation and migration by inactivation of p38 MAPK and JNK [109].

#### Prostate cancer

The lncRNA DRAIC was identified by RNA sequencing (RNA-seq) and was found to be downregulated in the progression from androgen-dependent to castration-resistant PCa. Moreover, higher levels of DRAIC prevent PCa invasion/migration by modulating androgen receptor (AR) and FOXA1 expression, resulting in longer DFS [14]. Similar to DRAIC, lncRNA PCAT29 acts as a tumor suppressor via modulation of AR [110]. Thus, these two lncRNAs may act as androgen-regulated tumor suppressors in PCa.

#### Neurospongioma

The lncRNA TSLC1-AS1 is the antisense transcript of tumor suppressor TSLC1. Its expression level is notably lower in glioma tissues. In glioblastoma U87 cells, the overexpression of TSLC1-AS1 upregulated TSLC1 and inhibited cell proliferation, migration, and invasion, while TSLC1-AS1 silencing has the opposite effects in human neuroglioma SNB-19 cells. These results suggest that TSLC1-AS1 may serve as a potential biomarker and therapeutic target for glioma by interfering with TSLC1 expression [111]. The lncRNA ADAMTS9-AS2 is the antisense transcript of tumor suppressor ADAMTS9. ADAMTS9-AS2 is also a glioma suppressor as expression is correlated with lower tumor grade and better prognosis. ADAMTS9-AS2 can modify malignant glioma behavior through DNA methyltransferase-1 [112].

LncRNA CASC2 (cancer susceptibility candidate 2) was originally described as a tumor suppressor gene in endometrial and colorectal cancers. Low expression levels have also been found in glioma tissues as well as in U251 and U87 glioma cell lines. Overexpression of CASC2 may hinder glioma progression by negative regulation of miR-21 [113].

#### Head and neck squamous cell carcinoma

Application of next-generation RNA-seq revealed 2808 differentially expressed lncRNAs between 40 head and neck squamous cell carcinoma and paired normal tissues. The expression levels of lncRNA LCE5A-1 and KCTD6-3 were markedly lower in head and neck neoplasm tissues and closely associated with the prognosis of patients. In head neck squamous cell carcinoma cells, overexpression of these two lncRNAs diminished cell growth and metastasis by regulating EMT-related gene expression and inhibiting tumor stem cell functions [114].

#### Renal cell carcinoma

The lncRNA growth-arrest-specific 5 (Gas5) sensitizes the cell to apoptosis by regulating the activity of glucocorticoids in response to nutrient starvation. Gas5 has also been linked to RCC as transcript levels are significantly reduced compared to unaffected normal renal tissues. Overexpression of GAS5 in A498 cells induced apoptosis and cell motility by acting as a transcription factor decoy for steroid hormone receptors [115]. LncRNA CADM1-AS1 expression is downregulated in tumor tissues of 64 patients with ccRCC, the most common RCC subtype, compared to adjacent non-tumor tissue. Furthermore, CADM1-AS1 expression was positively correlated with CADM1 mRNA expression in ccRCC specimens as well as 786-O and ACHN renal carcinoma cells. Thus, CADM1-AS1 was likely a ccRCC tumor suppressor that regulates cell proliferation, apoptosis, and migration via CADM1 [116].

#### Hepatocellular carcinoma and gastric cancer

The lncRNA PTENP1 modulates HCC cell behavior and gene networks by miRNA regulation, including the oncomirs miR-17, miR-19b, and miR-20a, as well as through modulation of the PI3K/AKT pathway. Overexpression of PTENP1 mitigated tumor growth, suppressed intratumoral cell proliferation, elicited apoptosis and autophagy, and inhibited angiogenesis in an animal model [117].

The lncRNA FENDRR controls the expression of target genes epigenetically by binding to PRC2. Low expression of FENDRR occured in GC cell lines and tissues and was associated with poor prognosis. FENDER overexpression suppressed invasion and migration in GC cells in vitro by attenuating FN1 and MMP2/MMP9 gene expression [118].

#### Pancreatic cancer

LncRNA ENST00000480739 expression is dramatically decreased in pancreatic ductal adenocarcinoma (PDAC), which contributed to tumor metastasis and progression by modulating HIF-1α. ENST00000480739 level was negatively related to tumor node metastasis stage and lymph node metastasis, indicating that it could be an independent prognostic factor of survival time in PDAC patients following surgery [119]. Further, enhanced ENST00000480739 expression in vitro inhibited PDAC cell invasion [119].

The lncRNA maternally expressed gene 3 (Meg3) was characterized as a tumor suppressor in pancreatic neuroendocrine tumor (PNET) cells. Overexpression of Meg3 in the insulin-secreting mouse PNET cell line MIN6 prevented proliferation and delayed cell cycle progression. Microarray studies have found that upregulated expression of Meg3 in MIN6 cells can attenuate the level of the proto-oncogene c-Met and reduce cell migration and invasion [120].

### Dual function of lncRNAs

Only a few of the lncRNAs characterized to date have shown dual tumor promotion and tumor suppressor functions. As mentioned earlier, the 693-bp lncRNA BANCR appears to both enhance and suppress metastatic potential. BANCR expression was elevated in GC tissues compared to matched non-cancerous tissue but downregulated in 113 NSCLC tumor tissues compared to match normal samples. Moreover, the aberrant expression of BANCR was positively associated with clinical stage, tumor depth, lymph node metastasis, and distant metastasis, and was an independent prognostic factor for GC/NSCLC in survival analysis [121, 122]. H19 has also been found to have both oncogenic and suppressive properties in different tumors [123]. While several studies reported that H19 functioned as an oncogenic lncRNA promoting tumor metastasis in GC, ovarian cancer, endometrial cancer, and bladder cancer, other studies found that it inhibits tumor metastasis via miR-675. Both H19 and H19-induced miR-675 expression level were significantly lower in metastatic PCa than less aggressive PCa. The H19/miR-675 axis may have diagnostic and therapeutic potential for advanced PCa cases [124]. Conversely, a miR-675 inhibitor or H19 siRNA dramatically increases HCC cell migration and invasion via the AKT/GSK-3β/Cdc25A signaling pathway [123]. The dual functionality of certain lncRNAs may stem from differential co-expression of other targets or tumor regulators in specific cell types. The ‘off-target’ effects of lncRNAs via indirect regulation or negative feedback look of gene-expression would be one potential reason.

## Conclusions and perspectives

At present, a large number of deregulated lncRNAs are involved in proliferation, apoptosis and cell-cycle control in the carcinogenesis, there is a growing list of lncRNAs that contribute to the specific process of metastasis. Some well-studied lncRNAs were demonstrated to be differentially expressed in metastatic tumor foci. In less than a decade of research on lncRNAs, as well as their prominent role in the regulation of tumor progression, have revealed the huge potential of the long non-coding transcriptome for therapeutic intervention, both as targets (when they are upregulated) and as drugs (when they are downregulated or lost). Yet in spite of lncRNAs becoming a research hotspot gradually, conceptual and technical limitations have restricted our deeper understanding on their contributions to human malignant tumors. Initially, the key factors for future development are to improve computational approaches and databases to distinguish potential functional and pathological links between lncRNAs and tumor occurrence and metastasis. In addition, transporting ncRNAs only to potentially cancerous cells is surely another main conundrum. It may be more complicate matters considering the size of lncRNAs when compared with miRNAs and siRNAs, bringing greater challenges such as delivery and stability to the target. However, nanoparticles and liposomes may be reconstructed in order to achieve tumor-targeting drug delivery, in an attempt to keep harmful side-effects remained manageable. Lastly, few animal models have been well developed to appraise the roles of lncRNAs in vivo and even as we know, there is still no lncRNA that enters clinical trials for the moment. The retardation of animal assays and human clinical trials could greatly impede the development and utilization of lncRNA-based new drugs and this will be another focus and difficulty of our research in the future.

Therefore, an absolute requirement is a more profound awareness of lncRNA functions and mechanisms, both in physiological and pathological circumstances. It is now necessary to expand and reconstruct lncRNA-enriched disease networks to develop new therapeutic strategies, allowing to increase the specificity and to decrease the toxicity especially in cancer therapy, so that lncRNA-based diagnostics and targeted therapeutics in cancer and metastasis can safely and successfully enter into regular clinical practice.

## Abbreviations

AKAP-9: PRKA kinase anchor protein 9
AR: Androgen receptor
BANCR: BRAF-activated non-coding RNA
BLBC: Base-like breast cancer
CASC2: Cancer susceptibility candidate 2
CCAT1: Colon cancer associated transcript 1
ccRCC: Clear cell renal cell carcinoma
CRC: Colorectal cancer
CUDR: Cancer-upregulated drug resistant
DFS: Disease-free survival
ECA: Esophageal adenocarcinoma
EMT: Epithelial-to-mesenchymal transition
EOC: Epithelial ovarian cancer
ERα: E2 receptor alpha
ESCC: Esophageal squamous cell carcinoma
EZH2: Enhancer of zeste homolog 2
FOX: Forkhead box
Gas5: Growth-arrest-specific 5
GBM: Glioblastoma multiforme
GC: Gastric cancer
GHSR: Growth hormone secretagogue receptor
GSCs: Glioblastoma stem cells
HCC: Hepatocellular carcinoma
HOST2: Human ovarian cancer-specific transcript 2
HOXC12: Homeobox C12
HOXD10: Hox transcription factor protein D10
LNAs: Locked nucleic acids
lncRNA: Long non-coding RNA
LSD1: Lysine-specific demethylase 1
MALAT1: Metastasis-associated lung adenocarcinoma transcript 1
Meg3: Maternally expressed gene 3
miRNA: microRNA
MMP-9: Matrix metalloproteinase-9
MMPs: Matrix metalloproteinases
NcRNA: Non-coding RNA
NSCLC: Non-small cell lung cancer
OS: Overall survival
OSCC: Oral squamous cell carcinoma
PCa: Prostate cancer
PCDHs: Cell adhesion molecules protocadherins
PCGEM1: Prostate cancer gene expression marker 1
PDAC: Pancreatic ductal adenocarcinoma
PNET: Pancreatic neuroendocrine tumor
PRC2: Polycomb repressive complex 2
PRG1: progesterone receptor 1
RACE: Rapid amplification of cDNA ends
RBM38: RNA binding motif 38
RCC: Renal cell carcinoma
RDA: RNA disruption assay
RNAi: RNA interference
TGF-β: Transforming growth factor beta
TSCC: Tongue squamous cell carcinoma
UCA1: Urothelial carcinoma-associated 1
VEGF: Vascular endothelial growth factor
WIF-1: Wnt inhibitory factor 1
